# Knowledge about Neonatal Danger Signs and Associated Factors among Mothers Attending Immunization Clinic at Arba Minch General Hospital, Southern Ethiopia: A Cross-Sectional Study

**DOI:** 10.1155/2019/9180314

**Published:** 2019-08-06

**Authors:** Nega Degefa, Ketema Diriba, Tekeste Girma, Amelework Kebede, Ayano Senbeto, Eyasu Eshetu, Zeleke Aschalew, Befikadu Tariku, Eshetu Zerihun

**Affiliations:** ^1^Nursing Department, College of Medicine and Health Sciences, Arba Minch University, Arba Minch, Ethiopia; ^2^Primary Health Care in Gamo Gofa Zone, Ethiopia; ^3^Department of Public Health, College of Medicine and Health Sciences, Arba Minch University, Arba Minch, Ethiopia

## Abstract

**Background:**

The first 28 days of life (the neonatal period) constitute the most vulnerable time for a child's survival. Overall 2.7 million neonatal deaths were stated by the 2015 global report of neonatal mortality and they account for 45% of under-five deaths. Sub-Saharan Africa remains the region with the highest risk of death in the first month of life and is among the regions showing the least progress in reducing neonatal mortality in the world. Ethiopia, as part of sub-Saharan Africa, also shares the greatest risk of neonatal death. A recent report in Ethiopia showed that neonatal mortality was 29 deaths per 1,000 live births. Therefore, the signs that suggest the onset of severe illness which leads to death and their contributing factors should be identified. The aim of the study was to assess knowledge about neonatal danger signs and associated factors among mothers attending immunization clinic at Arba Minch General Hospital.

**Method:**

Institution-based cross-sectional study design was employed from Feb to April 2018. Systematic sampling technique was used to select a total of 345 mother-child pairs. A pretested, structured, and interviewer-administered questionnaire was used to collect data. Data were entered using Epidata version 3.1 and analyzed using SPSS version 20. Bivariate and multivariable analysis were carried out using binary logistic regression to check and test the association between dependent and explanatory variables. Model fitness was checked by Hosmer-Lemeshow goodness of fit test.

**Result:**

Nearly two-fifths (40.9%) of all mothers had good knowledge about neonatal danger signs (95% CI; 35.7, 46.4). Close to thirty-three percent of mothers identified child's body hotness (fever) as a neonatal danger sign. Maternal educational status (AOR: 5.64; 95% CI: 1.68, 18.95) and attendance of postnatal care (AOR: 2.64; 95% CI: 1.36, 5.15) were significantly associated with maternal knowledge about neonatal danger signs in multivariable analysis.

**Conclusion:**

Even though considerable improvement has been achieved over the past decades as a result of expanded coverage of maternal and childcare services, still there are a significant number of mothers who have limited knowledge about neonatal danger signs. Therefore, interventional strategies that stress strengthening maternal education and ANC follow-up should be extended.

## 1. Background

The first 28 days of life (the neonatal period) constitute the most determinant time for a child's survival. In order to continue to accelerate the reduction in under-five mortality, focusing on newborns should be a primary concern [1]. Overall 2.7 million neonatal deaths were reported on the 2015 global report of neonatal mortality and they account for 45% of under-five deaths [2].

Worldwide, the neonatal mortality rate declined from 36 deaths per 1000 live births in 1990 to 19 deaths per 1000 live births in 2015 and the number of neonatal deaths decreased from 5.1 to 2.7 million. The remarkable decline in under-five mortality since 2000 has saved the lives of 48 million children under the age of five [3]. Regardless of the population growth in the developing countries, the number of deaths in under-five children has declined from 12.7 million in 1990 to almost 6 million in 2015 globally [4].

Sub-Saharan Africa (SSA) remains the region with the highest risk of death in the first month of life and is among the countries showing the least progress in reducing neonatal mortality in the world. However, Sub-Saharan Africa has also registered a significant improvement in reducing under-five mortality; its annual rate of reduction in neonatal mortality increased from 1.6% in the 1990s to 4.1% in 2000–2015. To hasten the progress of child survival rapidly in southern Asia and sub-Saharan Africa, due consideration should be given to ending preventable child deaths. One in 12 Sub-Saharan African child dies before the fifth birthday which is far beyond the average ratio of 1 in 147 in the high-income countries [3].

The recent report of Ethiopian Demographic and Health Survey (EDHS) showed that the rate of infant mortality was 20 deaths per 1,000 live births, while the overall under-5 mortality rate was 67 deaths per 1,000 live births and the neonatal mortality rate was 29 deaths per 1,000 live births. [5].

During each postnatal contact a child should be assessed for any of the danger signs and referred for additional evaluation if any of the following signs are present: stopped feeding well, history of convulsions or fits, fast breathing (breathing rate of ≥60 per minute), severe chest in-drawing, no spontaneous movement, fever (temperature ≥37.5°C), low body temperature (temperature <35.5°C), jaundice in first 24 hours of life, or yellow palms and soles at any age and signs of local infection: umbilicus red or draining pus, skin boils, or eyes draining pus. The family should be encouraged to seek healthcare early if they identify any of the above danger signs [6, 7]. Many approaches have been included in the health programs of Ethiopia to encourage early identification of neonatal health problems and management. Integrated Management of Newborn and Childhood Illness was one of these programs which focused on assessing neonatal danger signs and applies prompt treatment [6].

Maternal factors including young maternal age, primipara, short birth intervals, maternal health complications, and not breastfeeding and neonatal factors like preterm birth, low birth weight, multiple births, and male gender were associated with the death of newborn [2, 8–10]. Besides, factors like shortage of proper care during pregnancy, delivery, and the postpartum period and sociodemographic factors like residence and poor socioeconomic status were also associated with neonatal death [2, 11].

The causes of neonatal deaths and their management have been extensively studied, well understood, and described in the medical literature. However other equally important factors like maternal knowledge about neonatal danger signs (an abnormal health conditions which could eventually lead to life-threatening complications or death) have been given little attention and thus are not well studied or understood. Therefore, the aim of the study was to assess knowledge about neonatal danger signs and associated factors among mothers of under-12-month-old children visiting Arba Minch General Hospital for immunization service.

## 2. Methods and Materials

### 2.1. Study Setting and Period

The study was conducted at Arba Minch General Hospital which is found in Arba Minch city. Arba Minch town is a city and separate woreda located about 505km south of Addis Ababa, the capital city of Ethiopia. The city has a total population of 74,879, of whom 39,208 are men and 35,671 women. Gamo is the dominant ethnic group in the city and the majority of the residents were followers of Orthodox Christianity [12]. Education and health coverage of the town were 60% and 41%, respectively [13]. The antenatal coverage of at least four visits in the town was 38.5%. Institutional and skilled deliveries account for 21.8 and 16.4% in Arba Minch, respectively [14]. The area is well known for its banana, apple, and fish production which may affect child nutrition and health. We conducted a quantitative, institution-based cross-sectional study from February to April 15, 2018.

### 2.2. Population and Inclusion Criteria

The source population for this study was all mothers visiting immunization unit in Arba Minch General Hospital, whereas those mothers of under-12-month child visiting immunization unit at Arba Minch General Hospital during the study period and satisfying the inclusion criteria were the study population. All mothers who visited the immunization unit of Arba Minch General Hospital during the data collection period and had no serious illness or seriously ill child were illegible.

### 2.3. Sample Size and Sampling Procedure

The sample size for this study was calculated using the single population proportion formula by considering the following assumptions: The proportion of mothers who were knowledgeable about neonatal danger signs was 31.32% from the study conducted in Wolkite Ethiopia [15], with 95% confidence interval, 5% margin of error, and 10% nonresponse. A total of 364 mother-infant pairs were included in the study. The respondents were taken from Arba Minch General Hospital depending on the predetermined client flow rate. To recruit respondents for the interview, we identified the average number of clients visiting the immunization unit daily from client registration books or records for the last six months prior to data collection. Depending on this, we estimated the average client flow expected during the study period and it was 660. Then the average client flow during the study period was divided for the calculated sample size to identify the sampling interval and it was two. Thus, every second mother visiting the immunization unit was offered enrolment into the study.

### 2.4. Data Collection Method

The data was collected by interviewing mothers of under-12-month-old children visiting Arba Minch General Hospital for immunization service on exit using a pretested, structured, and interviewer-administered Amharic version questionnaire which was initially prepared in English. The questionnaire was adapted from prior studies conducted in different areas [16–19]. Three grade-twelve students who took an intensive two-day training on the data collection tool, fluently speak the local language, and were available throughout the data collection period collected the data. A trained and experienced clinical nurse supervised the data collection process on a daily basis. A pretest was done on 10% of the sample prior to the actual data collection and the result was used to correct wording, sequence, and approach of the questions.

### 2.5. Variables and Definition of Terms

The dependent variable is knowledge of mothers on neonatal danger sign (poor/good). The independent variable includes mothers age, mothers educational level, fathers educational level, ethnicity, mothers occupation, mode of delivery, place of delivery, and assistance during labor.

*Neonatal danger signs:* signs that indicate abnormal health condition and happen during the first 28 days of life [6].

*Knowledge:* mothers' level of awareness or mindfulness about neonatal danger sign. There were a total of 10 questions to assess mother's knowledge of neonatal danger sign and each right response was given a score of “1” while a wrong or unsure response was given a score of “0”. The total knowledge score ranges between 0 and 10.

*Good knowledge:* mothers who scored greater than or equal to the mean of total knowledge-based question.* Poor knowledge*: those mothers who have scored below the mean of total knowledge-based question. Hence those mothers who mentioned at least two neonatal danger signs were considered as having good knowledge about neonatal danger signs and mothers who mentioned less than two neonatal danger signs were considered as having poor knowledge.

### 2.6. Data Management and Analysis

The collected data were checked manually for completeness, coded, double entered using Epidata version 3.1, and then exported to SPSS version 20 for further analysis. Descriptive statistics like percentage and frequencies distribution were used to define respondents in relation to relevant variables and presented using tables and graphs. Crude odds ratio with 95% CI was computed to assess the association between each independent and the outcome variable (knowledge of mothers). Variables with a p-value less than 0.25 in bivariate logistic regression were entered into the multivariable analysis. Adjusted odds ratio with 95% confidence interval was used to assess the strength of association, and p-value < 0.05 was used to declare a statistical significance in the final model. Model fitness was checked by Hosmer-Lemeshow goodness of fit test.

### 2.7. Ethical Consideration

Ethical approval was obtained from Arba Minch University Ethical Review Board. An official letter from the university was submitted to Arba Minch General Hospital to obtain their permission. Then, informed verbal consent was take from each respondent before the commencement of the interview. The information obtained from them was kept confidential by not asking for identifying information.

## 3. Result

### 3.1. Sociodemographic Characteristics of the Mother

A total of 345 mother-infant pairs were enrolled in the study, giving a response rate of 95%. About forty-six percent of all mothers' age ranged between 25 and 34 years. Virtually all (97.7 %) of the respondents were married. Nearly two-fifths (39.7%) of respondents were from Gamo ethnic group and 45.2% of all respondents were followers of the protestant religion. About forty-one percent of all respondents' main occupation was a housewife. More than one-third (35.4%) of respondents' had completed primary education while 43.2% of their husbands had attended educational institution above secondary education (Table 1)

### 3.2. Mother's Healthcare Service Utilization Related Characteristics

Nearly ninety-six percent of respondents had antenatal care follow-up while they were pregnant for the index child. Almost half (50.4%) of the respondents had four visits whereas only 4.1% of them had no history of ANC follow-up at health facility. The majority (83.8%) of the respondents gave birth to the index child at a health facility. Eighty-four percent of respondents were assisted by a health professional during delivery. Most of the mothers (84.9 %) had no history of PNC follow-up and 56.2% of the mothers were multipara (Table 2).

### 3.3. Knowledge of Mothers about Neonatal Danger Signs

**Figure 1:**
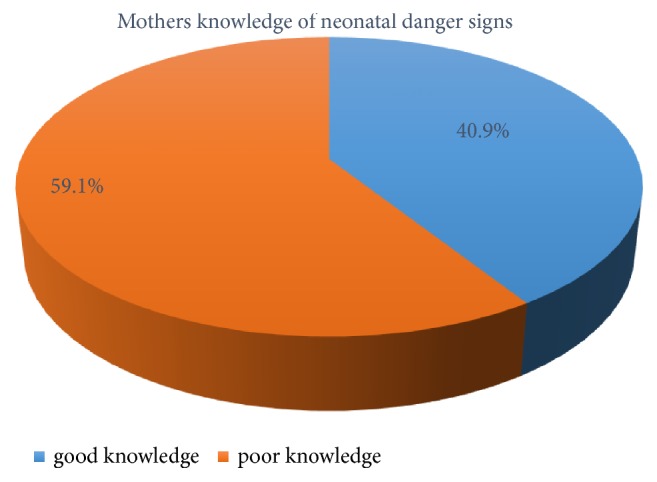
Knowledge of neonatal danger signs among mothers visiting the immunization unit of Arba Minch General Hospital, Southern Ethiopia, 2018.

Mother's knowledge of neonatal danger sign was assessed by listing the danger signs and letting them choose promptly. Then that sign which they thought a neonatal danger sign was selected from the given alternatives. Nearly two out of five (40.9%) of all mothers had good knowledge of neonatal danger signs (95% CI; 35.7, 46.4) (Figure 1).

The larger proportion of mothers (32.8%) recognized the child's body hotness (fever) as a neonatal danger sign. From the total of 345 surveyed mothers: 49(14.2%), 45(13.0%), 40(11.6%), 39(11.3%), and 36(10.4%) had stated stopped feeding on breast, difficulty of breathing, coldness, fast breathing, and no spontaneous body movement as neonatal danger signs, respectively. Only 26 (7.5%), 25 (7.2%), and 19 (5.5%) of mothers reported convulsion, jaundice, and any signs of local infection as neonatal danger sign whereas diarrhea was the most common non-WHO recognized neonatal danger sign noted by 40.9% of respondents (Table 3)

### 3.4. Factors Associated with Maternal Knowledge of Neonatal Danger Sign

In the bivariate logistic regression analysis, age of the mother, maternal educational status, father's level of education, parity, assistance during delivery, ANC follow-up, place of delivery, and marital status were associated with the dependent variable at p-value <0.25 and were included in the final model for multivariable analysis. In multivariable logistic regression mothers educational status and postnatal care follow-up were found to have a statistically significant association with maternal knowledge of neonatal danger signs. Hence, mothers who attend secondary educational level have 5.6 higher odds of having good knowledge of neonatal danger signs than those who had no formal education (AOR: 5.64; 95% CI: 1.68, 18.95). Likewise, odds of having good knowledge were 2.6 times higher among mothers who had postnatal care followup than those who failed to visit a health facility for PNC (AOR: 2.64; 95% CI: 1.36, 5.15) (Table 4).

## 4. Discussion

The current study showed that 40.9% of the women have a good knowledge regarding neonatal danger signs which is slightly lower than the finding of a study done in Chencha, Ethiopia which was 50.3% [20]. The difference may be attributed to the role of health extension worker in the dissemination of health information including newborn danger sign in the community and the difference in sample size.

The finding of the current study is higher than the finding of a study by Anmut and colleagues in Wolkite which was 31.32% [15]. The inconsistency may occur due to the gap in time between the two studies but the finding was consistent with the result of a study in Ghana [21].

Child's body hotness (fever) was the commonly recognized neonatal danger sign by 113 (32.8%) of the mothers in the current studies in Kenya [17] and in Nigeria [18] showing that 84.5% and 25.4% of mothers, respectively, identified body hotness as the leading neonatal danger sign. This may happen because most of the mothers can easily identify the febrile state in their child and the impending consequences.

In contrast to the finding of the current study, which mentioned diarrhea as the common non-WHO recognized danger sign, studies by Shally Awasthi et al. (north India) and Ekwochi et al. (Nigeria) identified excessive crying as a common non-WHO recognized danger sign among respondents [18, 22]. The difference may be due to the mother's level of perception about the severity of the problem which can in turn depend on the level of education and information about the issue.

Unlike the current study which identified a statistically significant association between mother's knowledge about neonatal danger signs and maternal sociodemographic factors, like maternal education and PNC follow-up, studies by Kibaru and Otara (Kenya) and Ekwochi et al. (Nigeria) showed no significant association with maternal sociodemographic variables considered in the study [17, 18].

Our study revealed that the mother's higher level of education and postnatal care follow-up were factors which had a statistically significant association with good knowledge of mother's about neonatal danger signs. Hence mothers who had a higher level of education had better knowledge about neonatal danger sign than those who had no formal education; likewise, mothers who had PNC follow-up have good knowledge about neonatal danger signs since they had increased chance of getting counselling about neonatal dangers sign. A study in northwest Ethiopia showed similar findings, in which the odds of having good knowledge were positively associated with mother's higher level of education (AOR = 3.41, 95% CI 1.37, 8.52) and attendance of postnatal care (AOR 2.08, 95% CI 1.22, 3.54) [19]. This may be justified by an increased chance of the mother's exposure to postnatal counselling which would possibly increase knowledge of the mother regarding neonatal danger signs. However, a study in Wolkite reported that factors like place of residence, presence of radio, and knowledge about essential newborn care had statistically significant associations with mother's knowledge about neonatal danger signs [15].

The main strength of this study was the application of the appropriate model for analysis. This study also had limitations like its cross-sectional nature which leads to the assessment of the exposure and outcome at the same point in time, so that we cannot formulate a cause and effect relationship between maternal knowledge about neonatal danger sign and the factors that were identified.

## 5. Conclusion

Despite the expanded coverage of maternal and child care services, many mothers have limited knowledge about neonatal danger signs which can cause the neonates to get delayed care. Mothers who had formal education and those who had postnatal care follow-up were more likely to have good knowledge about neonatal danger signs. Therefore, educating women can help tackle the effect of neonatal danger signs that indicate an abnormal health condition which ends in severe consequences.

This study thus suggests that as part of health education and sensitization, women should be taken through counselling on danger signs of newborn illness prior to their discharge from hospital so that they can easily detect signs and rush to healthcare facilities whenever necessary.

## Figures and Tables

**Table 1:** Sociodemographic characteristic of mothers (n=345) of under-12-month-old children visiting Arba Minch General Hospital for immunization services, Southern Ethiopia, 2018.

Variable	Category	Frequency (%)
Mothers age	15-24	70 (20.3)
	25-34	159 (46.1)
	35-44	116 (33.6)

Religion	Orthodox	126 (36.5)
	Protestant	156 (45.2)
	Muslim	57 (16.5)
	Catholic	6 (1.7)

Ethnicity	Gamo	137 (39.7)
	Gofa	59 (17.1)
	Zeise	23 (6.7)
	Wolayta	37 (10.7)
	Amhara	40 (11.6)
	Oromo	17 (4.9)
	Others	32 (9.3)

Marital status	Married	337 (97.7)
	Divorced/separated	4 (1.2)
	Widowed	4 (1.2)

Mother's educational status	No formal education	46 (13.3)
	Primary	122 (35.4)
	Secondary	91 (26.4)
	Above secondary	86 (24.9)

Mother's main occupation	Housewife	142 (41.2)
	Government employee	91 (26.4)
	Private employee	30 (8.7)
	Merchant	51 (14.8)
	others	31 (9.0)

Father's educational status	No formal education	34 (9.9)
	Primary	79 (22.9)
	Secondary	83 (24.1)
	Above secondary	149 (43.2)

**Table 2:** Healthcare service utilization related characteristics of mothers (n=345) of under-12-month-old children visiting Arba Minch General Hospital for immunization service, Southern Ethiopia, 2018.

Variables	Categories	Frequency (%)
ANC followup at health facility	Yes	331(95.9)
	No	14(4.1)

Number of ANC visits (n=331)	one	13(3.8)
	two	65(18.8)
	three	79(22.9)
	≥ 4	174(50.4)

Place of delivery	Home	56(16.2)
	Health facility	289(83.8)

Assistance during delivery	Health professional	290(84.1)
of the index child	Non-health professional	55(15.9)

PNC followup	Yes	52(15.1)
	No	293(84.9)

Parity	Primipara	151(43.8)
	Multipara	194(56.2)

**Table 3:** Prompt knowledge of neonatal danger signs among mothers (*n* = 345) visiting immunization unit of Arba Minch General Hospital, Southern Ethiopia, 2018.

Neonatal signs	Frequency (%)	
	No	Yes
Fever (body hotness)	232(67.2)	113(32.8)
Stopped feeding on breast	296(85.8)	49(14.2)
History of convulsion or fits	319(92.5)	26(7.5)
Jaundice (yellow palms and soles)	320(92.8)	25((7.2)
Fast breathing	306(88.7)	39(11.3)
Severe chest in drawing (difficulty of breathing)	300(87.0)	45(13.0)
Signs of local infection (red umbilicus or draining pus)	326(94.5)	19(5.5)
Coldness (hypothermia)	305(88.4)	40(11.6)
No spontaneous body movement	309(89.6)	36(10.4)
Diarrhoea	204(59.1)	141(40.9)

**Table 4:** Bivariate and multivariable analysis of factors associated with knowledge on neonatal dangers sign among mothers visiting immunization unit of Arba Minch General Hospital, Southern Ethiopia, 2018.

	Knowledge of mothers on danger sign		95% CI	
Variables	Poor (n (%)	Good (n (%)	COR	AOR
*Mother's age *				
15-24	47(67.1%)	23(32.9%)	1	
25-34	92(57.9%)	67(42.1%)	1.488(0.825,2.684)*∗∗*	1.643(0.857,3.149)
35-44	65(56.0%)	51(44.0%)	1.603(0.863,2.978)*∗∗*	1.536(0.764,3.089)
*Mother's educational status *				
No formal education	37(80.4%)	9(19.6%)	1	1
Primary	63(51.6%)	59(48.4%)	3.850(1.712,8.658) *∗*	4.454(1.423,13.936)*∗*
Secondary	42(46.2)	49(53.8%)	4.796(2.077,11.076)*∗*	5.638(1.677,18.949)
Above secondary	62(72.1%)	24(27.9%)	1.591(0.668,3.789)	1.832(0.520,6.456)
*Father's educational status *				
No formal education	26(76.5%)	8(23.5%)	1	1
Primary	45(57.0%)	34(43.0%)	2.456(0.989,6.094)*∗∗*	1.038(0.298,3.617)
Secondary	38(45.8%)	45(54.2%)	3.849(1.561,9.489)	1.198(0.324,4.428)
Above secondary	95(63.8%)	54(36.2%)	1.591(0.782,4.365)	0.750(0.198,2.841)
*Parity *				
Primipara	98(64.9%)	53(35.1%)	1	1
Multipara	106(54.6%)	88(45.4%)	0.651(0.421,1.009)*∗∗*	1.354(0.822,2.230)
*Assistance during delivery*				
Non-health professional	41(74.5%)	14(25.5%)	1	1
by health professional	163(56.2%)	127(43.8%)	2.282(1.192,4.369)*∗*	0.840(0.112,6.307)
*Place of delivery *				
Home	42(75.0%)	14(25.0%)	1	1
Health facility	162(56.1)%	127(43.9%)	0.425(0.222,0.813)*∗*	2.012(0.265,15.262)
*Antenatal care followup*				
Yes	192(58.0%)	139(42.0%)	4.344(0.957,19.718)*∗∗*	1.980(0.358,10.958)
No	12(85.7%)	2(14.3%)	1	1
*Postnatal care followup*				
Yes	24(46.2%)	28(53.8%)	1.858(1.026,3.365)*∗*	2.644(1.357,5.151)*∗*
No	180(61.4%)	113(38.6%)	1	1

*∗* P-value less than 0.05, *∗∗* p-value less than 0.25.

## Data Availability

The datasets used and/or analyzed during the current study are available from the corresponding author on reasonable request.

## Abbreviations

ANC: Antenatal care
PNC: Postnatal care
EDHS: Ethiopian Demographic and Health Survey
WHO: World Health Organization
SPSS: Statistical package for social sciences
AOR: Adjusted odds ratio
CI: Confidence interval.
